# Cross-Cultural Adaptation and Validation of a French Version of the Genetic Counseling Satisfaction Scale (GCSS) as an Outcome Measure of Genetic Counseling for Hereditary Breast and Ovarian Cancer

**DOI:** 10.3390/healthcare9091145

**Published:** 2021-09-02

**Authors:** Célia Villafane-Bernier, Julie Lapointe, Camille Raîche, Sophie Lauzier, Jocelyne Chiquette, Karine Bouchard, Sylvie Pelletier, Arian Omeranovic, Josée Rhéaume, Claire Brousseau, Johanne Hébert, Michel Dorval, Hermann Nabi

**Affiliations:** 1 Axe Oncologie, Centre de Recherche du CHU, Québec-Université Laval, 1050 Chemin Ste-Foy, Québec, QC G1R 3S3, Canada; celia.villafane-bernier.1@ulaval.ca (C.V.-B.); julie.lapointe@crchudequebec.ulaval.ca (J.L.); camille.raiche.1@crchudequebec.ulaval.ca (C.R.); sophie.lauzier@crchudequebec.ulaval.ca (S.L.); jocelyne.chiquette.med@ssss.gouv.qc.ca (J.C.); karine.bouchard@chudequebec.ca (K.B.); sylvie.pelletier833@videotron.ca (S.P.); arian.omeranovic@crchudequebec.ulaval.ca (A.O.); Michel.Dorval@crchudequebec.ulaval.ca (M.D.); 2 Faculté de Pharmacie, Université Laval, 1050 Av. de la Médecine, Québec, QC G1V 0A6, Canada; 3 CHU, Québec-Université Laval, 1050 Chemin Ste-Foy, Québec, QC G1S 4L8, Canada; josee.rheaume@chudequebec.ca (J.R.); claire.brousseau@chudequebec.ca (C.B.); 4 Centre de Recherche CISSS Chaudière-Appalaches, 143 Rue Wolfe, Lévis, QC G6V 3Z1, Canada; johanne_hebert@uqar.ca; 5 Département des Sciences de la Santé, Module des Sciences Infirmières, Campus de Lévis, Université du Québec à Rimouski (UQAR), 1595 Boulevard Alphonse-Desjardins, Lévis, QC G6V 0A6, Canada; 6 Département de Médecine Sociale et Préventive, Faculté de Médecine, Université Laval, 1050 Avenue de la Médecine, Université Laval, Québec, QC G1V 0A6, Canada

**Keywords:** cognitive interview, cross-cultural adaptation, French, genetic counseling, hereditary breast and ovarian cancer (HBOC), reliability, satisfaction measure, validation study

## Abstract

(1) Background: The Genetic Counseling Satisfaction Scale (GCSS) is a widely used tool to evaluate patient satisfaction. To our knowledge, a validated French-language version of this tool is not yet available. This article reports on the cross-cultural adaptation and validation of a French version of the Genetic Counseling Satisfaction Scale (GCSS) to evaluate genetic counseling services for patient consultation in hereditary breast and ovarian cancer (HBOC). (2) Methods: The scale was culturally adapted following guidelines from Beaton et al. (2000). Cognitive interviews were conducted to ensure items were understood according to the intended meaning. The internal consistency, floor and ceiling effects, and testing of group differences were assessed using a sample of 172 patients who attended a pretest group genetic counseling session. (3) Results: Participants understood all items according to the intended meaning. The internal consistency was high for the total scale (0.90) and for the corrected item-to-total correlations (varying between 0.62 and 0.78). No floor or ceiling effects were observed. Group difference analyses generally followed expectations. (4) Conclusion: This process generated a French version of the GCSS that is clearly understood by patients, and has psychometric properties adequately in line those reported for its original English version.

## 1. Introduction

Hereditary breast-ovarian cancer (HBOC) is among the most common hereditary cancer syndromes. Up to 10% of breast cancer cases are estimated to be inherited genetic variants in highly penetrant susceptibility genes [1,2]. For instance, carriers of pathogenic variants in the BRCA1 or BRCA2 genes have a 5- to 20-fold increased risk of developing breast or ovarian cancer [3,4]. This translates to an estimated cumulative lifetime risk of developing breast cancer at 72% for BRCA1 and 69% for BRCA2 pathogenic variant carriers [5]. The corresponding figures for ovarian cancer are 44% and 17%, respectively [5].

Individuals with a genetic/familial risk of a specific disease, including breast or ovarian cancer, may seek genetic counseling to obtain: medical information, support for genetic-testing decision-making, preventive screening, prophylactic or reproductive options, coping strategies, reduced uncertainty about risks, and facilitated family communication [6]. The pretest genetic counseling session aims to review and/or discuss topics such as family history, susceptibility genes, pros and cons of testing, family communication, and blood-collection logistics [7]. Genetic counseling professionals wish to provide services that meet or exceed patients’ expectations as well as the profession’s highest standards [7]. Given the value attached to the patient-centered approach, patient satisfaction regarding services is an important quality outcome. The systematic use of validated satisfaction scales, such as the Genetic Counseling Satisfaction Scale (GCSS), is a concrete way to monitor the attainment of this outcome [8].

The GCSS is an English self-reporting instrument developed by Tercyak et al. in 2001 [9] and validated by both Tercyak et al. in 2001 [9] and DeMarco et al. in 2004 [8] by patients attending a pretest genetic counseling session for fetal genetic anomalies or hereditary breast and ovarian cancer (HBOC), respectively. The GCSS is concise, simple to administer, and provides a global satisfaction score regarding the general goals of genetic counseling. The scale consists of six items that assess the process and content of counseling and is intended to be applicable to various contexts. The items provide an estimate of the patients’ level of agreement with statements about their genetic counseling experience. Response options follow a five-point Likert scale ranging from “1 = Strongly disagree” to “5 = Strongly agree”. To our knowledge, there is no validated version of the scale in the French language.

This paper presents the cross-cultural adaptation and validation of a French version of the Genetic Counseling Satisfaction Scale (GCSS) to evaluate genetic counseling services for patients consulting for hereditary breast and ovarian cancer (HBOC).

## 2. Material and Methods

### 2.1. Cross-Cultural Adaptation

The cross-cultural adaptation process of the scale followed Beaton’s guidelines [10]. Cross-cultural adaptation is a recognized multistep procedure that aims to obtain translated versions of questionnaires that achieve not only linguistic but also cultural equivalency with the original. This process comprises the steps of translation, synthesis, back translation, expert committee review, and pretesting to collect and verify the respondents’ understanding. For the latter step, we completed three cognitive interviews.

#### 2.1.1. Translation/Synthesis/Back Translation/Expert Committee Review

Two professional translators were independently asked to provide a French version of the GCSS. They were also asked to note their difficulties and hesitations, if any, in order to explain their decision-making process in choosing a proposed translation. Then, a conciliation meeting was held with the research team to discuss the inconsistencies and agree on a French version. This French version was then translated back to English independently by two other English-speaking professional translators who were blind to the original wording of the GCSS. This back translation enabled the research team to discuss minor discrepancies and agree on a preliminary French version. All steps of the process were coordinated by a research professional with a translation background (SP) who compared these different versions, summarized the outcomes, supported the consensus process, and revised the preliminary version. These working documents are available upon contacting the corresponding author.

#### 2.1.2. Cognitive Interviews

The first interview was conducted using the think-aloud method to identify terms or phrases found to be particularly problematic. The observations gathered from this first interview were verified in the other two interviews through a hybrid model approach (i.e., think aloud and probing method) [11]. There were three interviewers involved, one acting as a facilitator and two acting as note-takers. Since interviews were not recorded, in observance of our protocol approved by our Institutional Review Board, interviewers collected notes in a synchronous, shared document. The role of each interviewer was retained for all cognitive interviews. Following an introduction and an explanation of the interview goals, participants were invited to read the GCSS aloud. In the event of a misunderstanding, or a hitch, the interviewer would address and clarify with the participant any problems related to the wording of the item. A discussion with participants followed to find possible solutions to improve clarity (i.e., change a word, sentence re-wording, eliminate parts).

### 2.2. Validation

Inspired by the validation work undertaken for the original English version of the scale [8,9], the calculation of the scale’s psychometric properties included internal consistency, floor and ceiling effects, and testing of group differences on the total score for age, gender, marital status, childbearing, education, and cancer history. Based on the original validation work [8,9], we hypothesized that internal consistency would be high, the majority of participants would be highly satisfied, and that there would be no group differences on sociodemographic and medical variables. Similar to the original English version, we decided not to perform a factorial analysis to assess the dimensional structure of this French version of the scale.

### 2.3. Sample and Recruitment

All participants were from the Breast Disease Clinic of the CHU de Québec-Université Laval, Québec, Canada. The clinic has three referral indications: (1) being a member of a family where a mutation has been identified; (2) having a cancer history or diagnosis with indications for genetic testing; (3) having a family history with criteria for genetic testing [12]. Following approval from the CHU de Québec-University Laval Institutional Review Board (Project 2020–4695), we recruited participants to the cognitive interviews between October and November 2020. Participants had attended an online pretest group genetic counseling session less than a month prior to being invited by the research team. Participants were offered the choice to have their interview by teleconference or Zoom video conferencing platform. The validation sample (for the evaluation of psychometric properties of the scale) is comprised of all patients who completed the preliminary French version of the GCSS as part of the regular quality monitoring process immediately following their online pretest group genetic counseling session at the Breast Disease Clinic. The data were collected through the REDCap platform [13].

### 2.4. Data Analyses

Analysis of the cognitive interviews consisted of identifying elements that were problematic for the participants’ comprehensibility and grouping together common difficulties. The analysis was completed by two individuals (CVB and CR) trained in qualitative data analysis and overseen by a senior research professional with experience in performing cognitive interviews in the context of scale validation (JL). For the validation phase, the internal consistency was assessed with a raw and a standardized Cronbach’s alpha coefficient for the total score of the scale and for the influence of each item on the total scores [14]. The presence of floor and ceiling effects (i.e., ≥85% respondents choosing the lowest or highest response category [15]) was examined using descriptive statistics. Bivariate analyses were performed to determine whether there were group differences in satisfaction for any sociodemographic or medical factors.

## 3. Results

### 3.1. Cognitive Interviews

#### 3.1.1. Cognitive Interview Sample Characteristics

A total of six people were contacted, and three agreed to participate in a cognitive interview. The three participants were females, aged between 50 and 64 years old, and all had a history of cancer. They consulted following the recommendation of their treating physician and to understand the potential genetic risk for themselves and their relatives. At the time of enrolment, one participant had received her genetic test result, while the two others had not been tested. Two of these interviews were conducted by teleconference, and one was conducted via the Zoom video conferencing platform. All participants had already completed the French version of the GCSS a few days after their counseling session. All three participants had either French as their first language or were completely fluent in French.

#### 3.1.2. Response Analysis

All items of the French version of the GCSS were understood by participants according to their intended meaning. Only three suggestions of modifications (i.e., items 1, 3, and 5) were proposed. The term “stress” in item 1 was found by two participants to lack precision regarding the source of stress; the term “questionings” was suggested as a replacement. For item 3, two participants found the term “feel better” did not quite match the context of the pretest genetic counseling. The term “reassure” was proposed but was decidedly inappropriate since it is difficult to be reassured if someone has not yet received their genetic test result or has an unknown genetic variant. According to participants, “better informed” would be more adequate. The term “well-being” in item 5 was unclear to one participant, and the term “understanding” was proposed. Two participants were reluctant to answer item 4 since they found that a group session held online may not offer optimal freedom to express discomfort and concerns. Although we acknowledge and report the three suggestions, our expert review committee concluded that the preliminary French version that resulted from translation/back-translation by professionals was closer to the original scale and would ensure consistency of content while still maintaining patient understanding [10]. Consequently, we proceeded with the validation work with this French version. Details related to participants’ answers to the cognitive interviews are available upon contacting the corresponding author.

### 3.2. Validation

#### 3.2.1. Validation Sample Characteristics

A total of 454 patients were seen at the Breast Disease Clinic for a pretest genetic counseling group between August 2019 and February 2021. Of these, 176 (39%) completed the regular quality monitoring online follow-up survey that includes questions related to constructs such as satisfaction, knowledge, and perceived personal control. Answers from four patients were eliminated from the analyses because they did not complete all GCSS items, leaving a total of 172 patients. Demographic and medical characteristics of the validation sample are provided in Table 1. The vast majority of patients were between 36 and 69 years of age (79.1%), female (93.6%), living as a couple (75%), and had a University or post-secondary diploma (84.9%). The majority had children, and 73.6% had a history of cancer. The average total score for the French version of the GCSS was found to be at 26.8/30 (SD = 3.30), with an average item score of 4.5/5 (SD = 0.55).

#### 3.2.2. Internal Consistency

Since the raw and standardized Cronbach’s alpha coefficients yield very similar values (i.e., differing only on the second decimal), only the standardized coefficients are presented in Table 2. The Cronbach’s alpha coefficient for the total scale was found to be 0.88, and the corrected item-total correlations ranged from 0.62 to 0.79.

#### 3.2.3. Floor or Ceiling Effects

Even though the item’s response distributions were skewed towards positive answers, floor or ceiling effects were not observed with the French version of the GCSS (see Table 3 for details).

#### 3.2.4. Group Differences

Patients with a cancer history were slightly less satisfied with their pretest genetic counseling session than patients with no cancer history (26.4 vs. 28.0; t[168] = −3.52, *p* < 0.001). Patients with a university degree were slightly more satisfied than patients with a college degree or less (27.6 vs. 26.4; t[168] = −2.22, *p* < 0.03). Analyses showed no difference regarding satisfaction between those over 50 years of age and those under that age (26.8 vs. 28.8; t[168] = −0.13, *p* = 0.90). Finally, no differences in satisfaction were found regarding gender, marital status, and having children (data not shown but available on request).

## 4. Discussion

This study reports on a rigorous process undertaken to obtain a cross-cultural adaptation and validation of a French version of the GCSS for use with patients consulting in genetic counseling for HBOC. The process generated a French version that is clearly understood by patients and has psychometric properties that are in line with the ones reported for the original English version [8,9]. Indeed, we observed a Cronbach’s alpha coefficient of 0.88, while Tercyak (2001) and DeMarco (2004) reported values of 0.80 and 0.88, respectively. The overall satisfaction of our sample (i.e., 26.8/30) is also in the same range as previous work; 28/30 [9] and 26.9/30 [8]. Finally, like DeMarco (2004), we also observed no significant difference in satisfaction across age groups.

Unlike previous validation work, however [8], we did observe a small, yet statistically significant, difference in satisfaction according to previous cancer history, with patients with a cancer history being less satisfied with the genetic counseling session by 1.6 points on the 30-point scale. It is important to note that the DeMarco et al. (2004) study had a sample size of 61, while ours had a sample size of 172. It is possible that such a group difference appeared to be non-significant with a smaller sample. Several hypotheses could be generated to explain our group difference observation. It is possible that survivors of cancer, who have interacted extensively with different healthcare professionals, have gained knowledge about cancer and its familial and genetic implications. They may have derived less new knowledge about the genetic aspects of their condition during the counseling session and consequently judged it slightly less valuable to them. It is important to highlight that, with a score of 26.4/30, our sample of cancer survivors remained highly satisfied with the genetic counseling session.

Our observed group difference in patient education level contrasts with the absence of a difference observed on this aspect in the original scale [9]. Indeed, we observed that patients with a university degree were slightly more satisfied than patients with a college degree or less—for a total of 27.6/30 compared to 26.4/30. Such a difference may be explained by the fact that a lot of information is communicated at the pretest genetic counseling session. People with a university degree might be more prone to be highly satisfied with either a session heavy on new information or a session that builds on knowledge they already have. Although this difference is worth mentioning, a 1.2 point difference on a 30-point scale is not likely to have a significant impact on clinical practice. In addition, both of our groups remain highly satisfied, just like participants in the Tercyak et al. (2001) [9] study.

Through the conduct of our cognitive interviews, we collected suggestions of modifications for items 1, 3, and 5. One interesting comment was that the term “stress” was found by participants to be too strong to describe their feelings at their pretest genetic counseling session, which represents a concrete step in the process of genetic testing. Two participants mentioned that they had concerns and questions about genetic testing for cancer susceptibility, but although they understood the term “stress”, they reported that it was too intense to describe their situation. With the exception of genetic testing for Huntington’s disease, several systematic reviews concur that the genetic testing process might not be as stressful as medical and scientific communities initially feared [16,17,18]. As genetic testing and genomic medicine are finding their way into the mainstream of cancer care trajectories [19] and becoming recommended practice for an increasing number of chronic diseases, e.g., [20,21], feelings of stress may be even less frequent and intense in the future. Although the purpose of this work was to produce a reliable French version of the GCSS consistent with the original scale, and that we consequently chose to keep the term “stress”, it would be interesting in future work to compare the performance of the GCSS with a version where the term “stress” would be replaced by another less emotionally charged term.

The GCSS was said to be applicable to different genetic counseling practice contexts [8]. The GCSS has been used to evaluate genetic counseling for melanoma genomic risk [22], acute intermittent porphyria caused by mutations in the hydroxymethylbilane gene [23], prenatal genetic counseling [24], pancreatic cancer with the presence of CDKN2A [25], Lynch syndrome [26], coronary heart disease genomic risk [27], multiple sclerosis [28], Down syndrome [29] and breast and ovarian cancers [30]. However, the fact that the GCSS was deemed to be applicable to different counseling settings does not preclude the requirement to actually validate the scale in all its different usages [31]. To our knowledge, the GCSS has been validated in the context of fetal genetic anomalies and of hereditary breast and ovarian cancer and for an individual genetic counseling delivery method. Therefore, we believe it is important that the scale be validated for use in different medical conditions as well as different genetic counseling delivery methods and time points. For instance, our work is presently building evidence related to the reliability and validity of the scale for usage in group genetic counseling. Future validation work may reveal that some adjustments may be required before its use in other contexts.

### Strengths and Limitations

Considering that French is currently the 5th most widely spoken language worldwide [32], and that genetic information and counseling are increasingly part of mainstream oncology programs [19], we believe that French cross-cultural adaptation constitutes a significant contribution to the practice of genetic counseling and to the research efforts in this field. Any translation work bears a risk that meanings will be altered or lost. We have thus used a rigorous cultural adaptation process comprising translation and back-translation steps completed by cognitive interviews. Although it is recommended that large-scale national survey evaluation processes complete cognitive interviews in rounds of five to fifteen participants [33], there is no consensus about the adequate sample size for small and medium scale evaluations [34]. We believe our sample size of three was not a limitation in our cross-cultural adaptation work, even though we recognize that a diversity of respondents could have provided a variety of opinions on the possible difficulties or suggested changes. Our cognitive interviews were conducted in an environment of trust, thereby encouraging exchange and expression. Each of our participants understood the principles of the cognitive interview and felt at ease to discuss the clarity of the wording and propose adjustments. Finally, participants agreed on the possible modifications, which may indicate that the most important adaptation features of the scale were covered.

## 5. Conclusions

Satisfaction is important to monitor in order to maintain and improve the quality of services and experience for patients regarding content and delivery of genetic counseling. This study generated a French version of the GCSS that is clearly understood by patients, and has psychometric properties in line with the ones reported for its original English version. It would be important to pursue the validation work for this scale in other genetic counseling practice settings and delivery methods. Furthermore, future validation work should assess other psychometric properties such as test-retest reliability and factorial analysis to ensure the results are stable over a reasonable period of time and the items are aligned on only one construct as meant at the conception of the scale.

## Figures and Tables

**Table 1:** Characteristics of validation sample (*N* = 172).

Characteristics	*N* (%)
Age [missing values]	[2 (1.2)]
Less than 25	5 (2.9)
Between 26 and 35	9 (5.2)
Between 36 and 50	45 (26.2)
Between 51 and 69	91 (52.9)
70 and more	20 (11.6)
Gender [missing values]	[2 (1.2)]
Female	161 (93.6)
Male	9 (5.2)
Marital status [missing values]	[2 (1.2)]
Single	34 (19.8)
Living as a couple	129 (75.0)
Living with another adult	7 (4.0)
Children [missing values]	[2 (1.2)]
Has children	135 (78.5)
Has no child	35 (20.3)
Education [missing values]	[2 (1.2)]
High school or less	24 (14.0)
Non-university certificate or post-secondary diploma	90 (52.3)
University diploma	56 (32.6)
Personal cancer history [missing values]	[2 (1.2)]
No	45 (26.2)
Yes	125 (72.6)

**Table 2:** Reliability analysis and descriptive statistics.

Item	Item Wording	r *	Alpha If Item Deleted	Means (SD)
1.	Le professionnel en conseil génétique semblait comprendre le stress que je ressentais/*My genetic counselor seemed to understand the stresses I was facing*	0.67	0.86	4.35 (0.78)
2.	Le professionnel en conseil génétique m’a aidé(e) à déterminer ce que j’avais besoin de savoir pour prendre des décisions pour la suite des choses /*My genetic counselor helped me to identify what I needed to know to make decisions about what would happen*	0.71	0.85	4.70 (0.55)
3.	Je me suis sentie(e) mieux par rapport à ma santé après avoir rencontré le professionnel en conseil génétique /*I felt better about my health after meeting with my genetic counselor*	0.62	0.87	4.12 (0.88)
4.	La rencontre de conseil génétique a duré à peu près le temps dont j’avais besoin /*The genetic counseling session was about the right length of time I needed*	0.67	0.86	4.49 (0.67)
5.	Le professionnel en conseil génétique se préoccupait vraiment de mon bien-être /*My genetic counselor was truly concerned about my well-being*	0.78	0.84	4.48 (0.70)
6.	La rencontre de conseil génétique a été très utile pour moi/*The genetic counseling session was valuable to me*	0.66	0.86	4.64 (0.61)

*r:* corrected item-to-total correlations. * All corrected item-to-total correlations are significant at *p* < 0.0001.

**Table 3:** Item-level responses from GCSS to assess the presence of floor and ceiling effects (*N* = 172).

GCSS French-Version Items	Level *	*n* (%)
Le professionnel en conseil génétique semblait comprendre le stress que je ressentais/*My genetic counselor seemed to understand the stresses I was facing*	1	2 (1.2)
	2	2 (1.2)
	3	15 (8.7)
	4	67 (38.9)
	5	86 (50.0)
2. Le professionnel en conseil génétique m’a aidé(e) à déterminer ce que j’avais besoin de savoir pour prendre des décisions pour la suite des choses/*My genetic counselor helped me to identify what I needed to know to make decisions about what would happen*	1	1 (0.6)
	2	0 (0)
	3	2 (1.2)
	4	43 (25)
	5	126 (73.2)
3. Je me suis sentie(e) mieux par rapport à ma santé après avoir rencontré le professionnel en conseil génétique/*I felt better about my health after meeting with my genetic counselor*	1	4 (2.3)
	2	5 (2.9)
	3	18 (10.5)
	4	85 (49.4)
	5	60 (34.9)
4. La rencontre de conseil génétique a duré à peu près le temps dont j’avais besoin/*The genetic counseling session was about the right length of time I needed*	1	1 (0.6)
	2	2 (1.2)
	3	5 (2.9)
	4	67 (38.9)
	5	97 (56.4)
5. Le professionnel en conseil génétique se préoccupait vraiment de mon bien-être/*My genetic counselor was truly concerned about my well-being*	1	1 (0.6)
	2	2 (1.2)
	3	8 (4.6)
	4	64 (37.2)
	5	97 (56.4)
6. La rencontre de conseil génétique a été très utile pour moi/*The genetic counseling session was valuable to me*	1	1 (0.6)
	2	1 (0.6)
	3	3 (1.7)
	4	49 (28.5)
	5	118 (68.6)

* Patients were asked to indicate how much they agreed with each statement regarding their genetic counseling experience on a five-point Likert response scale (1 = strongly disagree to 5 = strongly agree).

## Data Availability

The data presented in this study are available on request from the corresponding author. The data are not publicly available due to privacy and ethical considerations.
